# Voice and Functional Outcomes of Transoral Laser Microsurgery for Early Glottic Cancer: Ventricular Fold Resection as a Surrogate

**DOI:** 10.14740/jocmr2216w

**Published:** 2015-06-09

**Authors:** Ilyes Berania, Christophe Dagenais, Sami P. Moubayed, Tareck Ayad, Marie-Jo Olivier, Louis Guertin, Eric Bissada, Jean-Claude Tabet, Apostolos Christopoulos

**Affiliations:** a Otolaryngology-Head and Neck Surgery Service, Universite de Montreal Hospital Center (CHUM), Montreal, Canada

**Keywords:** Transoral laser microsurgery, Tumor size, Functional outcomes, Quality of life

## Abstract

**Background:**

The aim of the study was to evaluate the oncological and functional outcomes with transoral laser microsurgery (TOLM) of patients with early glottic cancer.

**Methods:**

We have prospectively evaluated patients treated with TOLM for Tis, T1 or T2 glottic squamous cell carcinoma. Evaluation of oncological outcomes, and voice and functional outcomes was assessed using voice-handicap index 10 (VHI-10) and performance status scale for head & neck cancer patients (PSS-H&N). Predictors of poor voice quality were evaluated using Student’s *t*-test.

**Results:**

Thirty patients were included, with 17.7 months mean follow-up. There were no cases of locoregional recurrence. Twelve patients (40%) were considered as having a problematic voice outcome. Four subjects out of 30 (13.3%) had significant problems with understandability of speech. Significant differences (P < 0.05) in VHI-10 score were found with tumor stage and partial resection of the ventricular fold.

**Conclusions:**

We report excellent oncological and functional outcomes in early glottic cancer treated with TOLM, with advanced tumors and partial resection of the ventricular fold as a surrogate predicting worse voice outcomes.

## Introduction

Laryngeal cancer is the second most common head and neck malignancy, after oral cavity cancer [1], and the glottis is the most common site involved [2]. The treatment of early glottic cancer (T1 and T2) has been focused on maintaining optimal oncologic control as well as functional preservation of the larynx. Over the years, traditional open surgery has been replaced with less invasive procedures such as transoral laser microsurgery (TOLM) and radiotherapy, which have shown better functional results [3]. Recent studies suggest equal effectiveness for oncological control with both modalities [3]. However, limited information is available for comparative functional outcomes and quality of life (QOL) between these procedures [3]. The purpose of our study is to evaluate the oncological and functional outcomes with TOLM of patients with glottic cancer, and compare our data with functional outcomes of radiation-treated patients in the literature.

## Methods

We have prospectively evaluated 30 patients who underwent TOLM at the Universite de Montreal Hospital Center (CHUM), from January 2009 to February 2013. This study was approved by the institutional review board (Comite d’Ethique de la Recherche du CHUM). We have included all cases of T1 and T2 squamous cell carcinoma treated using TOLM at our institution during this time frame. There were no exclusions. Medical charts were reviewed for demographic characteristics (age, gender, tobacco exposure before and after treatment, and alcohol consumption), tumor characteristics (histology, TNM staging, and site and extension of the tumor) and treatment details (duration of anesthesia, presence of a pre-operative biopsy, partial resection of the ventricular fold, pathology results on margins, number of interventions, and type of resection). All surgeons are fellowship-trained head and neck oncologic surgeons with an experience of at least 60 transoral laser resections each,which is defined as an expert group [4]. Type of resection was determined using the European Laryngological Society classification (ELS, types I - V) [5].

Voice and functional outcomes were assessed using the voice-handicap index 10 (VHI-10) and the performance status scale for head & neck cancer patients (PSS-H&N). A single post-operative functional assessment (VHI-10 and PSS-H&N) at least 3 months postoperatively was conducted. The VHI-10 is a 10-item questionnaire which produces a total single score arrived at by a summation of scores allocated to different responses to items/questions that range from 0 to 40 points, with 0 indicating no disadvantage and 40 indicating maximum disadvantage [6]. Each item is answered using a five-point scale, with 0 indicating never and 4 always. The PSS-H&N is a clinician-rated assessment tool consisting of three subscales: 1) understandability of speech; 2) normalcy of diet; and 3) eating in public, which each produces a score from 0 (worst) to 100 (best) [7]. Oncological outcomes are locoregional tumor recurrence and survival rate.

A Student’s *t*-test was conducted to find significant associations between the surgical and demographic characteristics on functional outcomes. A P value < 0.05 was considered statistically significant.

In order to limit inter-examiner variability, a single person carried out both questionnaires via phone call. Each individual gave consent to the use of his or her personal information and results.

## Results

Demographic characteristics are presented in Table 1. We found a male to female ratio of 6. Medication count was used as a surrogate for comorbidities. Tumor characteristics are presented in Table 2. Treatment details are presented in Table 3. The majority of our patients underwent ELS type 2 cordectomy (53.6%). Patients were under anesthesia of an average of 91.3 ± 41.2 min.

**Table 1:** Demographic Results

	Number	Total %
Mean age (max, min)	68.2 (86, 54)	
Gender		
M	24	85.7
F	4	14.3
Smoking before treatment	19	67.9
Smoking after treatment	4	14.3
Alcohol*	15	53.6
> 3 medications	15	53.6

*Alcohol is defined as any alcohol consumption.

**Table 2:** Tumor Characteristics

	Number	Total %
Histology		
Squamous cell carcinoma	28	100
Pre-operative biopsy	25	89.3
Tumor staging (TNM)		
*In situ*	8	28.9
T1A	18	64.3
T1B	1	3.6
T2	1	3.6
N	0	0
M	0	0
Extension to the anterior commissure	1	3.6
Arytenoid extension	2	7.1
Subglottic extension	0	0
Supraglottic extension	0	0

**Table 3:** Treatment Characteristics

	Number	Total %
ELS type		
1	1	3.6
2	15	53.6
3	4	14.3
4	3	10.7
5a	3	10.7
5b	2	7.1
5c	0	0
Partial resection of ventricular fold	13	46.4
Positive pathology on specimen	19	67.9
Positive margins	0	0
Second look	8	28.9
Third look	2	7.1

Voice and functional outcomes are presented in Table 4. Voice results were determined using the VHI-10 questionnaire results. Problematic voice corresponds to a VHI-10 score higher than 11/40 [8]. Eleven patients (39%) were considered as having a problematic voice outcome. Functional outcomes were evaluated using the PSS-H&N questionnaire. Moderate to severe dysfunction of understandability, eating in public and normalcy of diet is considered when the PSS-H&N score was lower or equal than 50% [9]. Four subjects out of 30 (14.3%) had a problematic score in regards to problems with understandability. All patients scored 100% in “eating in public” and “normalcy of diet” categories. Mean follow-up time at evaluation was 17.3 months (SD: 9.4).

**Table 4:** Functional Outcomes

	Mean	Standard deviation
Follow-up (months)	17.3	9.4
Voice (VHI-10 score)	9.1	6.5
Understandability (PSS-H&N)	82.7	20.9
Normalcy of diet (PSS-H&N)	100	0
Eating in public (PSS-H&N)	100	0

No cases of locoregional recurrence were observed during the average 17.3 months follow-up. One case (3.6%) of non-cancer-related death was noted.

Predictors of voice quality are shown in Table 5. Significant differences (P < 0.05) in VHI-10 score were found with tumor stage and partial resection of the ventricular fold.

**Table 5:** Predictors of Voice Quality

	Mean VHI-10 scores	P value
Demographic characteristics		
Age		
≤ 65 years	8.8	0.851
> 65 years	9.3	
Gender		
M	13.0	0.820
F	9.8	
Smoking		
Smokers	6.3	0.169
Non-smokers	10.5	
Alcohol*		
No	9.6	0.704
Yes	8.6	
Tumor characteristics		
T staging		
*In situ*	4.8	0.004
1 or 2	11.9	
Surgical characteristics		
ELS		
1-3	7.6	0.102
4-5	12.6	
Partial resection of ventricular fold		
No	6.3	0.009
Yes	12.7	
Procedures		
1	10.5	0.055
> 1	5.9	

*Alcohol is defined as any alcohol consumption.

## Discussion

In this study, favorable oncological and functional outcomes were observed for patients with early glottic cancer treated by endoscopic laser resection. These findings support high efficiency of microsurgery as reported in previous studies. Local control rates and disease-specific overall survival in our study initially appear superior to past reports. In a meta-analysis, Higgins et al [10] demonstrated local control rates ranging from 75% to 93% for TOLM. Hartl et al [11] noted an overall local-recurrence-free survival rate of 89%, and disease-specific survival rate of 97% over a 5-year period. However, our study includes patients with shorter follow-up times and a higher proportion of early stage disease. Therefore, this may explain our higher control rates. In comparative studies, endoscopic laser surgery is thought to be as equally effective as radiotherapy in terms of oncologic control [12]. Multivariate analysis by Klintenberg et al [13] reported local control rates for glottic cancer treated primarily by radiotherapy of 90% for T1 tumors and 73% for T2 tumors. Kerr et al [14] noted no significant difference between the two modalities for disease-free overall survival (99±2% and 99±1%) and overall survival (91±3% and 90±4%) respectively.

Quality of voice is also comparable to studies in the literature. Peeters et al [15] reported abnormal VHI scores in 40% of patients with T1a glottic cancer after surgical resection, which is similar to our results. Functional outcomes measured with the PSS-H&N support that laser surgery mostly likely affects quality of speech and has minimal effect on public eating and normalcy of diet [16], which is also in line with our results. In a systematic review, Spielmann et al [17] included four studies using VHI score to compare voice quality after TOLM and radiotherapy. One study reported superior VHI scores with surgical resection, and no significant difference for the three remaining studies.

Our results show that partial resection of the ventricular fold results in poorer voice quality, which can be explained by the importance of this structure in influencing glottic aerodynamics [18]. Although intuitive, this point is rarely reported, and is important to address. Resection of the ventricular fold is most likely associated with larger tumors extending laterally that require wider exposure. Although tumor volume was not calculated in this study, it is generally accepted that smaller tumors treated with TOLM have better subjective voice outcomes [19]. Therefore, resection of the ventricular fold may be used as a surrogate of worse voice outcomes.

Stoeckli et al [20] reported better subjective scores with TOLM for swallowing solid foods (EORTC QLQ-H&N35 scores 24.4 vs. 5.0, P < 0.05). Moreover, a recent systematic review of functional outcomes after treatment of early glottic carcinoma has shown that none of the series included for review has evaluated swallowing function [17]. To our knowledge, no studies performing objective swallowing evaluations have been conducted, and this might be an interesting future direction in studies conducting head-to-head comparisons between the two treatment modalities.

Although current finding supports equal effectiveness of the two treatment options, additional benefits of transoral laser resection include shorter treatment time as a single outpatient procedure, reduced hospitalization and recovery time, and preservation of treatment options in case of recurrences [21, 22]. In addition, several studies have focused on the economic impact of glottic cancer treatment. The majority of findings report that TOLM is more cost-effective than radiotherapy [10, 23]. Phillips et al [24] demonstrated in a Canadian study, costs more than fourfold greater with radiotherapy than surgical treatment. These results would support surgical treatment for early glottic cancer over radiotherapy.

A notable limitation of this study is the small number of patients. In addition, it was not possible to directly compare patients treated by surgery and radiotherapy as patients are usually selected for radiotherapy in our center when they are deemed non-surgical due to increased comorbidities, which would have rendered groups non-comparable. Despite these limitations, this study allowed assessment of voice quality and functional outcomes with two validated questionnaires for all reported cases of early glottic cancer treated in our center.

Since most studies are retrospectively designed, definitive treatment guidelines for early glottic cancer remain disputable. Further randomized controlled trials comparing oncological and functional outcomes between laser surgery and radiotherapy may facilitate management of the disease.

### Conclusions

We report excellent oncological and functional outcomes in early glottic cancer treated with TOLM. Our results are similar to what has been previously reported in the literature, with more extensive resections, and partial resection of the ventricular fold as a surrogate for prediction of worse voice outcomes. It is an important point to remember when counseling patients on treating tumors with a lateral extent requiring partial resection of the ventricular fold.
